# Validation of the Donkey Pain Scale (DOPS) for Assessing Postoperative Pain in Donkeys

**DOI:** 10.3389/fvets.2021.671330

**Published:** 2021-06-11

**Authors:** Maria Gláucia Carlos de Oliveira, Valéria Veras de Paula, Andressa Nunes Mouta, Isabelle de Oliveira Lima, Luã Barbalho de Macêdo, Talyta Lins Nunes, Pedro Henrique Esteves Trindade, Stelio Pacca Loureiro Luna

**Affiliations:** ^1^Department of Animal Sciences, Semi-Árido Federal Rural University (UFERSA), Mossoró, Brazil; ^2^Department of Anatomy, Pathology and Veterinary Clinics, Federal University of Bahia (UFBA), Salvador, Brazil; ^3^Department of Veterinary Surgery and Animal Reproduction, School of Veterinary Medicine and Animal Science, São Paulo State University (UNESP), Botucatu, Brazil

**Keywords:** equines, castration, analgesia, welfare, animal behavior

## Abstract

This study aimed to validate a scale for assessing acute pain in donkeys. Forty-four adult donkeys underwent castration after sedation with intravenous (IV) xylazine, induction with guaifenesin and thiopental IV, local anesthetic block, and maintenance with isoflurane. The scale was constructed from a pilot study with four animals combined with algetic behaviors described for equines. After content validation, the scale was evaluated in 40 other donkeys by three blinded and one reference evaluator, by means of edited videos referring to the preoperative and postoperative periods: before anesthesia, 3–4 h after recovery from anesthesia, 5–6 h after recovery from anesthesia (2 h after analgesia with flunixin—1.1 mg/kg, dipyrone—10 mg/kg, and morphine—0.2 mg/kg) IV, and 24 h after recovery. Content validity, sensitivity, specificity, and responsiveness of behaviors were investigated to refine the scale. Intra- and inter-evaluator reliabilities were investigated by the weighted kappa coefficient, criterion validity by comparing the scale with the visual analog scale (VAS), internal consistency by Cronbach's α coefficient, item-total correlation by the Spearman coefficient, and intervention point for rescue analgesic by the receiver operating characteristics curve and Youden index. The scale showed very good intra-evaluator reliability (0.88–0.96), good to moderate (0.56–0.66) inter-evaluator reliability, responsiveness for all items, good criterion validity vs. VAS (0.75), acceptable internal consistency (0.64), adequate item-total correlation, except for head position and direction, and according to the principal component analysis, good association among items. The accuracy of the point for rescue analgesic was excellent (area under the curve = 0.91). The rescue analgesic score was ≥ 4 of 11 points. The scale can diagnose and quantify acute pain in donkeys submitted to castration, as the instrument is reliable and valid, with a defined intervention analgesic score.

## Introduction

Identifying alterations in pain behavior in donkeys is a difficult task for veterinarians, caregivers, and owners (1). An instrument that specifically diagnoses and measures acute pain is important, to provide a quick analgesic intervention to guarantee the welfare of these animals.

Unlike horses, the stoic behavior of donkeys, characterized by a lack of a clear expression of pain, coupled with poor knowledge of their normal habits, makes it difficult to understand their painful conditions and hamper the prompt establishment of adequate analgesic treatments. Therefore, pain scales developed for horses cannot be extrapolated for donkeys. In view of this, an ethogram, using surgical castration as a standard pain model (2), and other instruments have been developed to assess pain in this species (3, 4).

An accurate scale must demonstrate validity, reliability, sensitivity, specificity, and responsiveness by using a blind and random methodology. Validity is the ability of the instrument to evaluate what was proposed. Reliability is the consistency of results obtained by the same evaluators at different times or by different evaluators at the same time, to guarantee the consistency or stability of a measure. Responsiveness is related to the ability to identify changes in pain intensity over time or in response to analgesic intervention (5). A scale is specific and sensitive when a given pain behavior is absent when the animal is pain-free and present when the animal is suffering pain, respectively (6). A new proposed pain assessment instrument should undergo an in-depth statistical validation, as reported in cats (7), cattle (8), pigs (9), and sheep (10), to ensure its experimental and clinical applicability.

Two recently published donkey pain scales (Equine Utrecht University Scale for Donkeys Composite Pain Assessment—EQUUS-DONKEY-COMPASS and Equine Utrecht University Scale for Donkey Facial Assessment of Pain—EQUUS-DONKEY-FAP) have shown excellent intra- and inter-evaluator reliability (3). Although both instruments were responsive (their pain scores were greater in donkeys suffering pain vs. pain-free donkeys), the limitation mentioned by the authors include the fact that the study was unblinded, possibly generating expectation bias. The first shown instrument is time consuming and none of them were submitted to an item-by-item analysis for refinement and a thorough validation. A Grimace scale has also been proposed for donkeys post castration, but only its sensitivity, specificity, and accuracy were analyzed (4), therefore deserving a further appraisal.

In view of the above, the objective of the present study was to investigate repeatability, reproducibility, sensitivity, specificity, content validity, construct validity (responsiveness), concurrent and predictive criterion validity, internal consistency, item-total correlation, and cut-off point for intervention analgesia of a pain scale in donkeys submitted to orchiectomy. This study is a follow up of the previous study that developed an ethogram after orchiectomy (2).

## Materials and Methods

The study was approved by the Ethics Committee on Animal Use of UFERSA (Semi-árido Federal Rural University) under protocol 23091.011744/2017-59. Animals from an Animal Protection Association (APA) in the region were used. The person responsible for the APA signed an informed consent and authorized the procedures and publication of the results.

### Design and Experimental Protocol

The study was conducted at the University Veterinary Hospital. A total of 44 male donkeys of the breed Northeastern, approximate age 6.4 ± 3.1 (2–14) years, weighing 120 ± 13 kg (87–133 kg) were used. For inclusion, the animals were required to allow human approach and placement of a halter, be considered healthy after a complete clinical examination, and present normal laboratory examinations (blood count, urea, creatinine, aspartate aminotransferase, alanine aminotransferase, and total proteins). The animals were dewormed with ivermectin, vaccinated against rabies, and housed in groups of five in 10 × 7 m outdoor paddocks, with shade. The donkeys were fed on Napier grass (*Pennisetum purpureum)* and concentrate (milled corn, soybean meal, wheat bran, common salt, and calcitic limestone) twice a day and drinking water *ad libitum*. The period of acclimatization to the new environment was 4 weeks. The same surgeon performed orchiectomy on the animals in the morning. Postoperative assessment was performed on the same day and on the following morning.

In a pilot study, analysis of the normal behaviors of the species and possible behaviors related to pain was performed in four animals. These donkeys, as well as those used in the main study, were housed in individual covered 2 × 4 m stalls 24 h before the beginning of the study, where they were intermittently filmed and continuously monitored by an internal video circuit, before castration and for up to 24 h after the procedure. The anesthetic protocol was 0.5 mg/kg of intravenous xylazine (IV), followed by anesthetic induction with 100 mg/kg of guaifenesin associated with 5 mg/kg thiopental IV. The anesthesia was maintained with isoflurane with a flow of 10 ml/kg/min of O_2_. A local anesthetic block was performed with 2% lidocaine in each spermatic funiculus and incision line, totalizing a volume of 15 mL per testis. Throughout the procedure and until the first signs of recovery (before positioning in sternal decubitus) the animals were monitored for non-invasive blood pressure, heart and respiratory rates, and temperature, using a multiparameter monitor (Dixtal 2021, Dixtal Biomédica, São Paulo, Brazil). At the end of the surgery, the animals received anti-tetanus serum and 30,000 IU/kg of sodium penicillin intramuscularly. Food (bulky) and water were offered 2 h after an anesthetic recovery. Recovery time was recorded from the end of isoflurane administration until the donkeys stood up.

All donkeys received postoperative analgesia consisting of flunixin meglumine 1.1 mg/kg, dipyrone 10 mg/kg, and morphine 0.2 mg/kg, applied IV 4 h after recovery from anesthesia and repeated every 24 h for 3 days (11). The surgical wound was treated for the same period with silver sulfadiazine. The films were analyzed for 30 min in each period: 24 and 16 h before and 1, 2, 4, 5, 8, and 24 h after recovery from anesthesia in order to record behaviors to construct an ethogram, using continuous recording and animal focal sampling methods (12). The ethogram data were published in a parallel study (2) to identify pain behaviors associated with surgical castration in donkeys. Donkeys were placed back with their group 3 days after castration.

In order to test the validity of the content, a list of behaviors was produced by combining the ethogram developed in the pilot study, reports in the literature on donkey pain-related behaviors (1, 2, 13–15), and the authors' experience. This list was sent to three evaluators with knowledge on equine behavior, who independently analyzed and scored each item according to the degree of importance regarding the behavior to detect pain into −1 = irrelevant item; 0 = not known; 1 = relevant item. Subsequently, the values were added and divided by the number of experts. Items that achieved a score ≥ 0.5 and were reported as pain-related behaviors according to the literature in donkeys (1, 2, 13–15) were included on the pre-refinement scale to be analyzed by the evaluators. These methods to check content validity have been described elsewhere (8, 11, 16–19) (Table 1).

**Table 1:** Criteria used to select the behaviors included in the donkey pain scale used for video analysis, based on content validity, and behaviors reported in the literature.

**Behavior category**	**Ev. 1**	**Ev. 2**	**Ev. 3**	**Content validity**	**Behavior reported in the literature**
Position in stall	1	1	−1	0.33	(1)
***Response to opening of the stall door***	**1**	**0**	**1**	**0.66**	(11)
Response to evaluator approaching	1	1	−1	0.33	(11)
**Posture (stand up or lay down)**	**1**	**1**	**1**	**1**	(1, 20)
***Head position***	**1**	**1**	**1**	**1**	(2, 11)
**Locomotion when stimulated by the evaluator by using a halter to lead**	**1**	**1**	**1**	**1**	(21–24)
***Ear position***	**1**	**1**	**1**	**1**	(1, 25, 26)
Ear Height (Up or down)	1	1	−1	0.33	(1)
***Head direction***	**1**	**1**	**1**	**1**	(1, 25, 26)
Head movement	1	1	−1	0.33	(6)
Tail position	1	0	0	0.33	(1, 2, 11)
**Appetite for food offered**	**1**	**1**	**1**	**1**	(11, 25, 26)
***Response to palpation of the wound***	**1**	**1**	**1**	**1**	(6, 11)
Response to auditory stimulus (palm clap)	1	1	−1	0.33	(11)
Sweating	1	−1	1	0.33	(6)
***Miscellaneous Behaviors***
**Rolls**	**1**	**1**	**1**	**1**	(1)
**Lies down and stand up**	**1**	**1**	**1**	**1**	(1)
**Digs**	**1**	**1**	**1**	**1**	(1)
**Kicks abdomen**	**1**	**1**	**1**	**1**	(27–29)
***Lifts pelvic limb***	**1**	**1**	**1**	**1**	(1, 2)
**Exposes penis**	**1**	**1**	**1**	**1**	(11)
Lifts pelvic limb and extend head	1	−1	−1	0.33	(11)
**Shakes tail**	**1**	**1**	**1**	**1**	(1, 25, 26)

*Ev., Evaluator; each item was classified as relevant (+1), not known (0), or irrelevant (−1), the values were added and divided by the number of evaluators; items with values of content validation < 0.5 were not included in Table 2 (scale used for video analysis). In bold are the items approved in the content validation, as the mean score was ≥ 0.5 and because the specific pain-related behavior had been reported in the literature in horses or donkeys; in italics are the behaviors that remained on the scale after refinement (DOPS)*.

The proposed instrument gave rise to a variable score scale composed of behavioral categories, each with three ordinal descriptive levels, scored from zero, reflecting a state of normality, to one and two, reflecting moderate and intense pain state, respectively, with a maximum score of 23 points (Table 2).

**Table 2:** Acute pain scale used for the analysis of videos of donkeys submitted to castration before refinement.

**Category**	**Description**	**Score**
Posture	Standing up	0
	Lying in sternal decubitus	1
	Lying in lateral decubitus	2
Head position	Above the withers or eating	0
	At the height of the withers	1
	Below the withers but without eating	2
Head direction	Straight head or eating	0
	Turned to environmental stimuli and not to body parts	1
	Looks at the affected area	2
Ear position	Forward facing predominantly	0
	Lateral facing predominantly	1
	Backward facing predominantly	2
Miscellaneous Behaviors	Rolls	1
	Lies down and stand up	1
	Digs	1
	Kicks abdomen	1
	Lifts one of the pelvic limbs	1
	Exposes penis	1
	Shakes tail	1
Response to opening the stall	Moves toward the door or is close to the door	0
	Looks at the door but does not move toward the door	1
	Does not respond to door opening	2
Locomotion when stimulated by the evaluator by using a halter to lead	Moves around freely alone	0
	Does not move, or is reluctant to move	1
	Agitated, restless	2
Appetite (food offered by the evaluator)	Moves toward the food and eats	0
	Hesitates to move toward the food, but eats	1
	Does not show interest in food, does not eat	2
Response to palpation of the wound	No response or alteration in relation to the moment before surgery	0
	Mild response to palpation of the surgical wound	1
	Violent response to palpation of the surgical wound	2
Total		23

For the application of the scale, 40 donkeys were castrated under the same anesthetic protocol and monitored by video as described previously by using two cameras positioned on opposite sides, at the top of each stall. The videos were watched for 1 h and carefully edited by the in-person evaluator to produce films lasting 3–4 min at the following moments: before castration (M0), between 3 and 4 h after recovery from anesthesia (M1), between 5 and 6 h after recovery from anesthesia, 2 h after analgesia, using the same protocol described in the pilot study (M2) and 24 h after recovery from anesthesia (M3). The video editor selected the behaviors observed in 1 h to include in videos of 3–4 min.

Videos were recorded by the in-person evaluator performing the following sequence of actions in the four moments previously mentioned: (1) opened the stall door and observed the animal; (2) entered the stall and approached the animal slowly; (3) placed the halter and stimulated the animal to walk; (4) offered food (Napier grass); (5) palpated the area of the surgical wound.

The order of the edited videos was randomized for donkeys and time-points (randomizer.org) and watched by three blinded evaluators, and the in-person evaluator, experienced in equines. After a 30-day interval, the videos were rearranged into a new random sequence and watched by the same evaluators for a second time (2nd evaluation phase).

The evaluators received the following instructions: Watch the video and answer: (1) does the animal require administration of analgesics according to your clinical experience? (2) complete the visual analog scale (VAS—from 0, no pain, to 100 mm, worst possible pain), numerical (NS—from 0, no pain, to 10, worst possible pain), and simple descriptive scales (SDS—from 0, without pain, to 3, severe pain) (30, 31); (3) complete the proposed scale by selecting the descriptor level within each item that best represents what you observed; (4) if in doubt, watch the video again.

### Statistical Analysis

Statistical analyses were performed using R software in the integrated development environment RStudio (Version 1.0.143— 2009-2016, RStudio, Inc.), and an α of 5% was considered.

#### Reference Evaluator

The reference evaluator was the investigator directly involved with the practical work, i.e., donkey management, in-person pain assessment, and data recording and editing. The reference evaluator data from the second evaluation phase were used for calculations of sensitivity and specificity, construct validity of each item of the scale, concurrent criterion validity, internal consistency, item-total correlation, and principal component analysis.

#### Sensitivity and Specificity

A test is sensitive when it expresses a high true positive rate to detect a present disorder or to detect what it is supposed to measure. The test is specific when it expresses a high true negative rate; in this case the test should not detect the disorder when the disorder is absent. Therefore, M1 scores were used to test sensitivity, since at this moment, the donkeys should express pain and would be considered true positives. Likewise, M0 scores were used to test specificity, since at this moment, the donkeys should be free of pain and would be considered true negatives (16).

The M1 scores were transformed into dichotomous factors: level “0”—the absence of pain behavior for a given item; levels “1” and “2”—the presence of pain behavior. Sensitivity was determined by the formula *S* = *TP*/(*TP* + *FN*), where S = sensitivity, TP = true positive (scores representing pain behaviors 1 or 2 at the time when the animals should present pain since it was after the surgery), FN = false negative (scores that represented behaviors of the absence of pain 0 at the same moment above).

The scores at M0 were transformed into dichotomous factors. The specificity was determined by the formula Sp = *TN*/(*TN* + *FP*), where Sp = specificity, TN = true negative (scores that represented behaviors of absence of pain 0 at the moment when the animals should supposedly not present pain, as it was before the surgery), FP = false positive (scores that represented pain behaviors 1 or 2 at the same moment above).

#### Construct Validity

To evaluate the responsiveness of the instruments, the scores of each item of the proposed scale and NS, SDS, VAS, and the indication for rescue analgesic according to the evaluators' clinical experience over time (M0 *vs*. M1 *vs*. M2 *vs*. M3) were compared using data from the second evaluation phase of the reference evaluator. Responsiveness was also calculated for the total score of the proposed scale for each evaluator. For the dichotomous variables, we applied logistic regression analysis using the *post-hoc* Tukey test. The normality of each variable at each moment was evaluated by boxplots, histograms, and the Shapiro-Wilk test. All variables were non-parametric; therefore, we used the Friedman test in which the *p-*value was corrected with the Bonferroni procedure.

Items showing either specificity or sensitivity > 70% and responsiveness were included in the definitive scale (6, 10) and submitted to the following analyses after refinement of the scale.

#### Repeatability and Reproducibility

The repeatability of the evaluators' responses, related to the level of agreement of each evaluator with themself, was measured with the first and second evaluation phases of the videos. For the reproducibility, the level of agreement between the reference evaluator and the other three evaluators was estimated in the second evaluation phase. The weighted kappa coefficient was used (*k*_*w*_) to calculate the agreement of the indication for rescue analgesic according to the evaluators' clinical experience and of each scale item score, NS, and SDS (32). Disagreements were weighted according to the distance to the square of perfect agreement. The confidence interval (CI) of 95% of the *k*_*w*_ was estimated (33). The intraclass correlation coefficient (ICC) type “agreement” and its 95% CI were used for the VAS (34). For the sum of the proposed scale, the ICC type “consistency” and its 95% CI were used.

#### Criterion Validity

For concurrent criterion validity, the sum of the proposed scale *vs*. NS, SDS, and VAS was correlated by the Spearman rank correlation coefficient (*r*_*s*_) (8, 11, 17). A further test of concurrent criterion validity was to calculate the agreement between the reference and the other evaluators (8, 11, 17).

Predictive criterion validity was assessed by the number of donkeys that should receive rescue analgesia according to the Youden Index (described below) in the moment of greatest pain (M1) (10).

#### Internal Consistency

The consistency of each scale item score at each moment of pain evaluation was estimated by Cronbach's alpha coefficient (α) (35). The internal consistency was considered as follows: 0.60–0.64, minimally acceptable; 0.65–0.69, acceptable; 0.70–0.74, good; 0.75–0.80, very good; and > 0.80, excellent (36).

#### Item-Total Correlation

The item-total correlation was investigated to assess the homogeneity of the scale. Spearman's non-parametric coefficient was used to correlate each item with the sum of all scale items, removing the score of that item. The item-total correlation of each item with the total score should range between 0.30 and 0.70 (16).

#### Principal Component Analysis (PCA)

Association of items with each other at all moments together was assessed by PCA, based on the scores of the 2nd evaluation phase of all evaluators (37). Load values ≥ 0.50 or ≤ −0.50, in representative dimensions, suggested a significant association among items (eigenvalue> 1 and variance > 20%) (38).

#### Cut-Off Point for Rescue Analgesic

The minimum score related to the indication of the analgesic intervention was determined by analyzing the receiver operating characteristic (ROC) curve, using all the moments of evaluation of the pain of the proposed scale scored by all evaluators in the second evaluation phase. The need for analgesia according to the clinical experience, after the evaluators had watched the videos, was used as the true value and the total score of the Donkey Pain Scale (DOPS) as a predictive value to build a ROC curve. The area under the curve (AUC) indicates the discriminative ability of a test (8, 17, 39). The graphical representation of the relation between the “TP” (*S*) and “FP” (1-*Sp*) is the Youden Index (*YI*) = point of greatest sensitivity and specificity simultaneously, expressed by the formula:

$$\begin{array}{l} YI\;=\;\left(S\;+\;Sp\right)-1 \end{array}$$

The diagnostic uncertainty zone, used to define the diagnostic accuracy, was the highest interval of one of these two methods: (i) the 95% confidence interval replicating the original ROC curve 1,001 times by the bootstrap method or (ii) the interval between the sensitivity and specificity values of 0.90 (10).

Based on the number of donkeys with total pain scores above the Youden index, a score indicative of rescue analgesia before (M0) and after surgery (M1) was used to calculate the specificity (M0) and sensitivity (M1) of the scale to identify truly negatives (pain-free) and truly positives (suffering pain) donkeys. The frequency of scores greater than the Youden index should be very low in M0 (no pain) and very high in M1 (the most intense pain).

## Results

The results were obtained from the castration of 40 Northeastern donkeys. The procedure lasted on average 34 ± 6 min and the animals were standing 45 ± 7 (38–52) min after the anesthesia.

### Content Validity

Table 2 contains the items included in the scale after the content validation (Table 1). A score of 0 indicated normality or no change, 1 moderate change, and 2 marked change.

### Sensitivity and Specificity

The sensitivity and specificity ranged from excellent (95–100%) to not sensitive or specific (<70%) (6) (Table 3). Ear position was both sensitive and specific. The ears' lateral or backward position was specific and sensitive, in a situation of pain, while in animals without pain, the ears turned forward. Response to the door opening and to palpation of the wound, and miscellaneous behaviors (Table 2; rolls, lies down and stand up, digs, kicks abdomen, lifts one of the pelvic limbs, exposes penis and shakes tail) were sensitive to detect animals with pain, but not specific since they could be present in animals without pain. Head position and direction and locomotion when led were specific but were not sensitive. The appetite for food offered by the evaluator did not demonstrate sensitivity or specificity.

**Table 3:** Specificity and sensitivity of the donkey acute pain scale before refinement.

**Behavior category**	Specificity^*^	Sensitivity^*^
*Response to the opening of the stall door*	0.62	**0.98**
Posture	NA	NA
*Head position*	**0.85**	0.50
Locomotion when stimulated by the evaluator by using a halter to lead	**0.80**	0.30
*Ear position*	**0.72**	**0.88**
*Head direction*	**0.87**	0.43
Appetite for food offered	0.47	0.68
*Response to palpation of the wound*	0.65	**0.83**
*All miscellaneous behaviors grouped (below)*	0.50	**0.88**
*Lifts pelvic limb*	**0.97**	0.58
*Exposes penis*	**0.72**	0.03
*Shakes tail*	0.2	0.65

*In italics are the behaviors that remained on the scale after refinement. In bold are the values with specificity or sensitivity ≥ 70%*.

* *classification of specificity and sensitivity: excellent 95–100%; good 85–94.9%; moderate 70–84.9%, not sensitive or not specific <70% (6); NA, not determined, since the animals were always standing. M1 scores were used to test sensitivity (donkeys feeling pain—true positives) and M0 scores were used to test specificity (donkeys free of pain—true negatives)*.

### Construct Validity

Tables 4 and 5 present the data regarding the responsiveness of the scales. The postoperative scores of all items that remained after refinement (Table 6) increased significantly before analgesia when compared to the other time-points for all evaluators. The indication of the need for analgesia was higher at M1 > M3 > M0 = M2.

**Table 4:** Median and amplitude of the indication for rescue analgesic and scores of the Donkey pain scale (DOPS) (pre- and after refinement) and unidimensional scales to evaluate acute pain in 40 donkeys submitted to orchiectomy, over time.

**Behavior categories**	**M0 Med (Min–Max)**	**M1 Med (Min–Max)**	**M2 Med (Min–Max)**	**M3 Med (Min–Max)**
Posture	0 (0–0)	0 (0–0)	0 (0–0)	0 (0–0)
**Head position**	**0**^**b**^ **(0–0)**	**0.5**^**a**^ **(0–2)**	**0**^**b**^ **(0–1)**	**0**^**b**^ **(0–2)**
**Head direction**	**0**^**b**^ **(0–1)**	**0**^**a**^ **(0–2)**	**0**^**b**^ **(0–2)**	**0**^**a**^ **(0–2)**
***Ear position***	***0**^**c**^**(0–1)***	***2**^**a**^**(0–2)***	***0**^**c**^**(0–2)***	***1**^**b**^**(0–2)***
Miscellaneous behavior	0.5^bc^ (0–2)	1^a^ (0–2)	0^c^ (0–2)	1^ab^ (0–2)
Exposes penis^*^	11	1	4	8
Lifts pelvic limb^*^	1	23	1	6
Shakes tail^*^	31	26	28	33
**Lifts pelvic limb**	**0**^**b**^ **(0–1)**	**1**^**a**^ **(0–1)**	**0**^**b**^ **(0–1)**	**0**^**b**^ **(0–1)**
**Response to opening the stall door**	**0**^**b**^ **(0–2)**	**2**^**a**^ **(0–2)**	**0**^**b**^ **(0–2)**	**1**^**b**^ **(0–2)**
Locomotion when led	0^ab^ (0–1)	0^a^ (0–1)	0^b^ (0–1)	0^ab^ (0–1)
Appetite for food offered	1^ab^ (0–2)	1^a^ (0–2)	0^c^ (0–1)	0^bc^ (0–1)
***Response to palpation of the wound***	***0**^**c**^**(0–2)***	***1**^**a**^**(0–2)***	***0**^**c**^**(0–2)***	***1**^**b**^**(0–2)***
**Total sum of scale pre-refinement (****Table 2****)**	**2.5**^**c**^ **(0–7)**	**8**^**a**^ **(1–12)**	**2**^**c**^ **(0–5)**	**5**^**b**^ **(0–10)**
**Total sum of scale after refinement (****Table 6****)**	**1**^**c**^ **(0–5)**	**6**^**a**^ **(1–9)**	**1**^**c**^ **(0–4)**	**3**^**b**^ **(0–6)**
Numerical scale	1^c^ (1–3)	5^a^ (1–7)	1^c^ (1–2)	3^b^ (1–5)
Simple descriptive scale	1^c^ (1–2)	2^a^ (1–3)	1^c^ (1–2)	2^b^ (1–3)
VAS	0^c^ (0–38)	45.5^a^ (0–71)	0^c^ (0–25)	27^b^ (0–51)
Indication for rescue analgesic	0^c^ (0–1)	1^a^ (0–1)	0^c^ (0–1)	1^b^ (0–1)
Frequency of rescue indication^**^	8	29	7	11

*The comparisons were performed with logistic regression for the need for rescue analgesic and the Friedman test for the other variables (p < 0.05). In bold and italic are the items that differentiated between moderate and intense pain; In bold are items that remained in the scale after refinement; Med, Median; Min, Minimum; Max, Maximum; M0, before castration; M1, between 3 and 4 h after recovery from anesthesia; M2, between 5 and 6 h after recovery from anesthesia, 2 h after analgesia; M3, 24 h after recovery from anesthesia*;

abc *lowercase letters indicate significant difference between moments, being a>b>c*;

* *number of animals that presented the behavior*;

** *number of YES responses to the indication for rescue analgesic according to the clinical experience (of the total of 40 donkeys)*.

**Table 5:** Median (amplitude) total scores of the DOPS from all evaluators.

	**M0**			**M1**			**M2**			**M3**		
**Evaluator**	**Med**	**Min**	**Max**	**Med**	**Min**	**Max**	**Med**	**Min**	**Max**	**Med**	**Min**	**Max**
01	1^c^^B^	0	6	5^a^^B^	1	9	2^bc^^AB^	0	7	3^b^	0	8
02	2^c^^AB^	0	4	6^a^^AB^	3	10	2^c^^AB^	0	4	4^b^	1	6
03	2.5^c^^A^	0	7	5^a^^B^	1	8	2^bc^^A^	0	5	3^b^	0	6
04^*^	1^c^^B^	0	5	6^a^^A^	1	9	1^c^^B^	0	4	3^b^	0	6

abc *Lowercase letters indicate a significant difference between moments, being a>b>c*;

AB *capital letters significant difference between evaluators at each moment*;

* *reference evaluator; Med, median; Min, minimum; Max, maximum*.

**Table 6:** Definitive DOPS (Donkey pain scale).

**Category**	**Description**	**Score**
Head position	Above the withers or eating	0
	At the height of the withers	1
	Below the withers but without eating	2
Head direction	Straight head or eating	0
	Turned to environmental stimuli and not to body parts	1
	Looking at the affected area	2
Ear position	Forward facing predominantly	0
	Lateral facing predominantly	1
	Backward facing predominantly, rigidly	2
Lift pelvic limb	The animal lifts one of the pelvic limbs	1
Response to opening the stall door	Moves toward the door or is close to the door	0
	Looks at the door but does not move toward the door	1
	Does not respond to door opening	2
Response to palpation of the affected area	No response or alteration in relation to the moment before pain	0
	Mild response to palpation of the affected area	1
	Violent response to palpation of the affected area	2
**Total**		**11**

**Figure 1:**
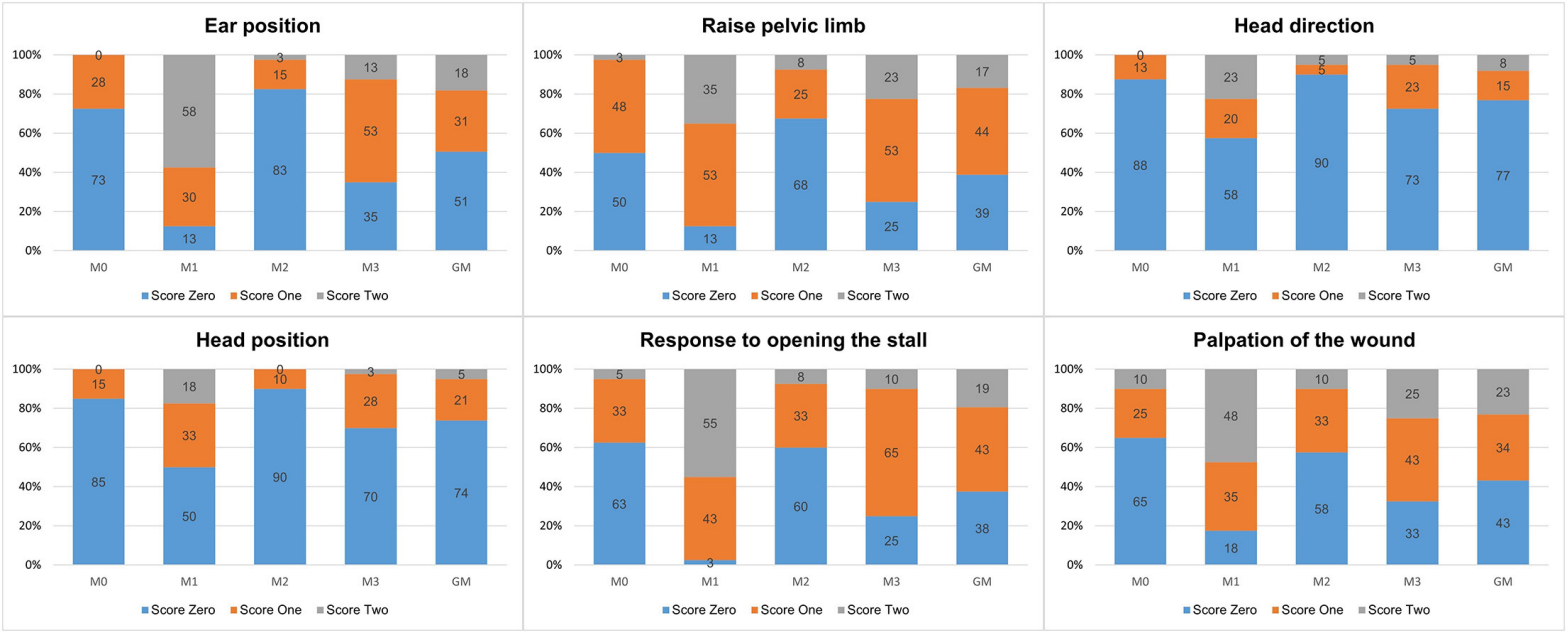
Frequency (%) of pain scores, for the DOPS to evaluate acute pain in donkeys.

The frequency of occurrence of pain score levels for each item at each moment and all moments together is presented in Figure 1. Except for head position and head direction, scores 1 and 2 predominated in donkeys feeling pain at M1 and M3. There was greater presence of score 2 in M1 than in M3, when donkeys were suffering maximum and moderate pain, respectively.

As described in the method section, the behaviors showing either specificity or sensitivity <70% and no responsiveness were excluded from the subsequent calculations. The following analysis was performed only for the definitive DOPS (Table 6).

### Repeatability

The intra-evaluator repeatability was very good for all scales (Table 7), except for the indication of rescue analgesic according to the evaluators' clinical experience, considered good (40). The evaluator with the best reliability was the reference evaluator (Ev. 4—in-person evaluator).

**Table 7:** Repeatability (intra-rater reliability) of the DOPS and unidimensional scales to evaluate pain in donkeys submitted to orchiectomy.

**Categories**	**Ev. 1 Weighted Kappa (Min–Max)**	**Ev. 2 Weighted Kappa (Min–Max)**	**Ev. 3 Weighted Kappa (Min–Max)**	**Ev. 4 (RE) Weighted Kappa (Min–Max)**
**Head position**	**0.95 (0.77–0.93)**	**0.96 (0.91–1.00)**	**0.82 (0.74–0.91)**	**0.88 (0.80–0.95)**
**Head direction**	0.78 (0.69–0.88)	0.74 (0.65–0.84)	**0.90 (0.83–0.96)**	**0.93 (0.88–0.97)**
**Ear position**	**0.82 (0.74–0.89)**	**0.87 (0.81–0.93)**	**0.93 (0.89–0.97)**	**0.89 (0.84–0.93)**
Lift pelvic limb^*^	0.51 (0.36–0.67)	0.31 (0.17–0.45)	0.62 (0.49–0.76)	**1.00 (1.00–1.00)**
**Response to opening of the stall door**	**0.86 (0.79–0.92)**	**0.97 (0.94–1.00)**	**0.94 (0.90–0.98)**	**0.81 (0.74–0.88)**
**Response to palpation of the wound**	**0.91 (0.86–0.95)**	**0.97 (0.94–1.00)**	**0.93 (0.89–0.97)**	**0.92 (0.88–0.96)**
Total score of DOPS^**^	**0.91 (0.88–0.93)**	**0.88 (0.84–0.91)**	**0.93 (0.91–0.95)**	**0.96 (0.94–0.97)**
Indication of analgesia?	0.74 (0.63–0.84)	0.66 (0.54–0.78)	0.67 (0.55–0.79)	**0.90 (0.83–0.97)**
**NS**	**0.89 (0.94–0.95)**	**0.91 (0.89–0.94)**	**0.87 (0.83–0.89)**	**0.93 (0.91–0.95)**
**SDS**	**0.88 (0.85–0.92)**	**0.81 (0.75–0.86)**	0.74 (0.66–0.81)	**0.86 (0.81–0.92)**
VAS^***^	**0.90 (0.87–0.93)**	**0.92 (0.90–0.94)**	**0.90 (0.86–0.92)**	**0.93 (0.90–0.95)**

*Ev., Evaluator; RE, reference evaluator; Min–Max, Minimum–Maximum; In bold are the values with very good correlation. Interpretation: very good 0.81–1.0; good: 0.61–0.80; moderate: 0.41–0.60; reasonable: 0.21–0.4; poor < 0.2 (40)*;

* *included separately after refinement*;

** *intraclass correlation coefficient*;

*** *intraclass agreement coefficient*.

### Reproducibility

The agreement level between reference and “blinded” evaluators 1 and 2 was good for DOPS and moderate for the other scales and indication of rescue analgesia. Evaluator 3 had the worst reproducibility results (moderate for DOPS and reasonable for other scales) (Table 8).

**Table 8:** Reproducibility (inter-rater reliability) of the DOPS and unidimensional scales to evaluate pain in donkeys submitted to orchiectomy.

**Categories**	**RE *vs*. Ev. 1 Mean weighted Kappa (Min–Max)**	**RE *vs*. Ev. 2 Mean weighted Kappa (Min–Max)**	**RE *vs*. Ev. 3 Mean weighted Kappa (Min–Max)**
Head position	0.25 (0.07–0.43)	0.30 (0.15–0.45)	0.16 (0.01–0.31)
Head direction	0.24 (0.08–0.40)	0.11 (0.01–0.21)	0.29 (0.13–0.46)
**Ear position**	**0.54 (0.43–0.64)**	0.37 (0.22–0.51)	0.23 (0.08–0.38)
Lift pelvic limb^*^	**0.68 (0.54–0.82)**	**0.50 (0.34–0.65)**	**0.51 (0.35–0.67)**
**Response to opening of the stall door**	0.32 (0.20–0.45)	**0.44 (0.31–0.57)**	**0.41 (0.29–0.53)**
**Response to palpation of the wound**	**0.49 (0.36–0.61)**	**0.48 (0.36–0.60**)	**0.43 (0.30–0.57)**
Total score of DOPS^**^	**0.66 (0.57–0.74)**	**0.65 (0.55–0.73)**	**0.56 (0.44–0.66)**
**Indication of analgesia?**	**0.50 (0.37–0.63)**	**0.47 (0.33–0.61)**	0.29 (0.15–0.44)
**NS**	**0.57 (0.48–0.66)**	**0.54 (0.42–0.66)**	**0.46 (0.34–0.58)**
**SDS**	**0.44 (0.33–0.55)**	**0.43 (0.32–0.54)**	0.37 (0.25–0.49)
VAS^***^	**0.43 (0.30–0.55)**	**0.50 (0.37–0.60)**	0.40 (0.26–0.52)

*RE, reference evaluator (Ev. 4); Ev., Evaluator; Min–Max, Minimum–Maximum; In bold are items with correlation varying from moderate to incredibly good. Interpretation: very good 0.81–1.0; good: 0.61–0.80; moderate: 0.41–0.60; reasonable: 0.21–0.4; poor < 0.2 (40)*;

* *included after refinement*;

** *intraclass correlation coefficient*;

*** *intraclass agreement coefficient*.

### Criterion Validity

Considering all the moments, the correlation between the total DOPS score and SDS, NS, and VAS was 0.68, 0.77, and 0.76, respectively, confirming criterion validity (16).

Criterion validity was also checked by reproducibility compared to the reference evaluator (Table 8). In this case, the correlations among evaluators were moderate for RE *vs*. Ev. 3 and good for RE *vs*. Ev. 1 and 2.

According to predictive criterion validity, agreement, and coherence suggestive of the necessity for rescue analgesia based on the Youden index were similar among evaluators (Table 9). Between 80% (Ev. 3) and 98% (RE) of donkeys would receive rescue analgesia in the moment of pain (M1), and between 3 and 10% would receive unnecessary analgesia in M0, showing that the scale presented both sensitivity to detect pain in donkeys suffering pain and specificity to detect in pain-free animals.

**Table 9:** Number/percentage of donkeys with scores ≥ 4 (Youden index) indicative of rescue analgesia before and after surgery.

	**Number of donkeys with rescue analgesia score (≥** **4)**			
**Evaluation period**	**Evaluator 1**	**Evaluator 2**	**Evaluator 3**	Evaluator 4^*^
M0 (Specificity)	4 (10%)	1 (3%)	4 (10%)	3 (8%)
M1 (Sensitivity)	34 (85%)	36 (90%)	32 (80%)	39 (98%)

* *Reference evaluator. M0, before castration; M1, between 3 and 4 h after recovery from anesthesia, before analgesia*.

### Internal Consistency and Item-Total Correlation

The internal consistency through the Cronbach's alpha coefficient considering all moments grouped was 0.64, therefore minimally acceptable (36). Except for head position and direction, item-total correlation of all other items *vs*. the total score ranged between the acceptable values 0.30 and 0.70 (16) (Table 10).

**Table 10:** Internal consistency and item-total correlation of the DOPS.

**Category**	**Internal consistency (Cronbach's α coefficient)**	**Item total (Spearman's coefficient)**
All items	0.64	–
	Excluding each item separately	
Head position	0.63	0.27
Head direction	0.64	0.26
Ear position	0.52	**0.56**
Lift pelvic limb	0.60	**0.41**
Response to opening the stall	0.60	**0.41**
Response to palpation of the wound	0.59	**0.43**

*Interpretation of Spearman's item-total correlation (rs)—bold values between 0.3 and 0.7 are accepted [16]. Cronbach's α coefficient was calculated by the total score of the scale and excluding each item from the scale. Interpretation: minimally acceptable 0.60–0.64, acceptable 0.65–0.69, good 0.70–0.74, very good 0.75–0.80, and excellent > 0.80 [26]*.

### Principal Component Analysis (PCA)

Only one dimension of the PCA of DOPS demonstrated a representative eigenvalue (> 1) and variance (> 20). Other items than head direction showed a significant association between them (load values > 0.50) (Table 11).

**Table 11:** Load, eigenvalue, and variance of the DOPS.

**Items**	**Dimension 1**
Head position	**−0.59**
Head direction	0.27
Ear position	**0.73**
Lift pelvic limb	**0.67**
Response to opening the stall	**0.52**
Response to palpation of the wound	**0.56**
**Eigenvalue**	**1.98**
**Variance**	**33.05**

*Principal component analysis in representative dimensions (eigenvalue > 1 and variance > 20%). Bold values indicate items with a load value ≥ 0.50 or ≤ −0.50 showing an association between them*.

### Point for Rescue Analgesic

**Figure 2:**
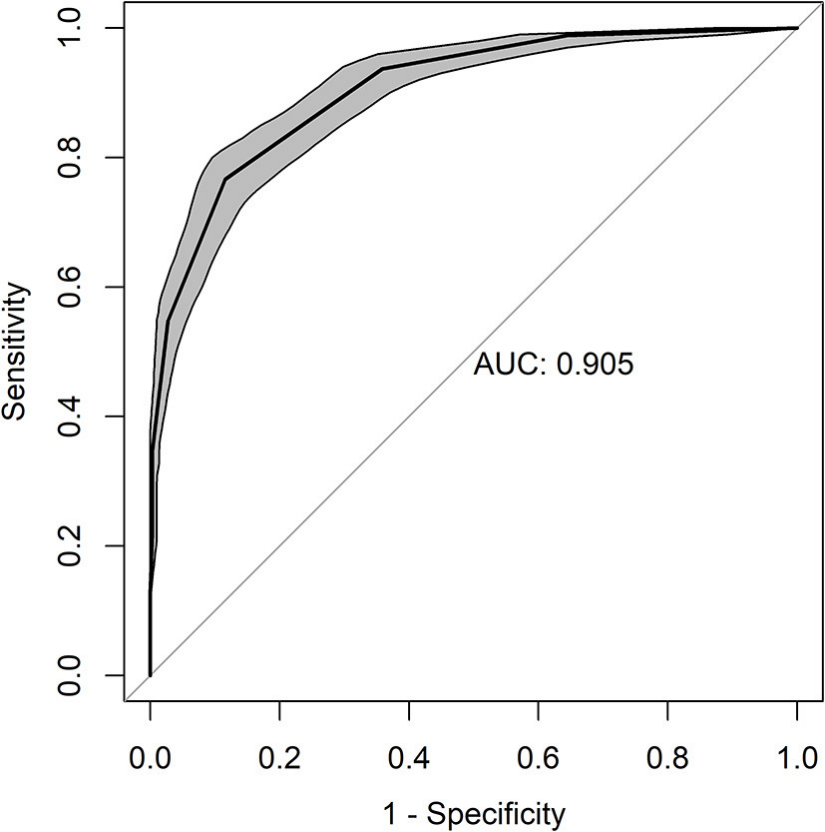
ROC (receiver operating characteristic) curve for the DOPS. Area under the curve (AUC) of 0.905.

**Figure 3:**
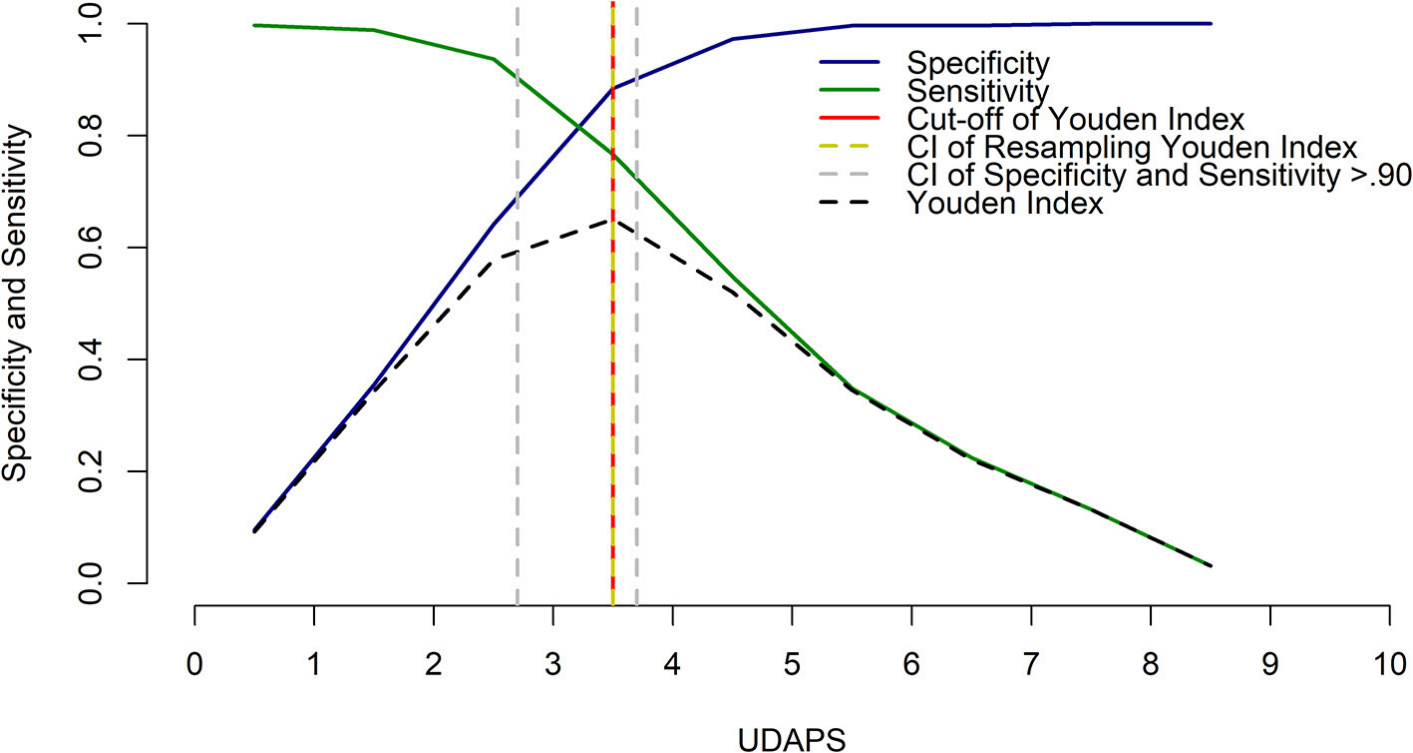
Two-graph ROC curve for the DOPS with the Youden index and diagnostic uncertainty zone of the cut-off point based on the confidence interval (CI) between the sensitivity and specificity values of 0.90.

Through analysis of the ROC curve, the area under the curve was 0.91 (Figure 2), which indicates the high accuracy of the rescue point (41). Based on the highest value of sensitivity and specificity (Figure 3), the cut-off point ≥ 4 was identified (range 0–11 points), with a sensitivity of 77% and a specificity of 88%. The resampling confidence interval for the Youden index was 3.5. The interval between the sensitivity and specificity values of 0.90 was between 2.7 and 3.7. Based on the last result, the scores of the diagnostic uncertainty zone ranged from 3 to 4; <3 indicates pain-free donkeys (true negatives) and > 4 indicates donkeys undergoing pain (true positives).

## Discussion

This study determined a quantifiable method to diagnose pain in donkeys, involving behavioral alterations after a standardized nociceptive stimulus producing mild to moderate visceral pain. The proposed scale for evaluating acute postoperative pain in donkeys presented responsiveness, since it identified animals with different degrees of pain, including moderate pain 24 h after surgery, and indicated a point of analgesic intervention that can be used in clinical situations. This is one of the most relevant points in the validation of an animal pain scale, as reported for other species (8–10, 17). Thus, the tool identifies and quantifies the presence of pain in this normally unrecognized species, guaranteeing the decision making for the provision of analgesia and, consequently, the donkeys' welfare.

Although content validation is limited to the subjective experience of each evaluator, behaviors that were not approved at this stage were also not observed in the ethogram of these animals (2). An exception was the auditory stimulus response, which was not evaluated, since this could frighten the animals, causing stress and interfering in the evaluation of other behaviors. Although not part of the measurements, the donkeys did not approach the evaluator in the in-person observations and did not move their heads, common attitudes in horses (6, 11, 42). The statistical criteria used for content validation in the present study have been reported previously (8, 11, 16–19); other measures incorporating pain-related behaviors in donkeys described in the literature were also employed to guarantee good coverage of relevant behaviors in the instrument (1, 6, 11, 20–23, 25, 26).

The overall scores, the response to the opening of the stall door, the head and ear position, head direction, reaction to the palpation of the wound, and lift pelvic limb were responsive to identify donkeys without pain *vs*. donkeys suffering pain, and a reduction in pain score after analgesia. Donkeys with pain interact little with the environment (15), as they did not react to the evaluator's entrance in the stall and remained with their head low but without eating. Otherwise in horses (11), the response to opening the stall door was not relevant in identifying pain. The head's direction was specific, and results were similar to those observed in horses with abdominal pain and after castration (11, 27). Looking at the affected region is also a characteristic behavior of acute pain in donkeys after castration in the present study.

Donkeys always remained standing; therefore, the posture was not modified to assess responsiveness, sensitivity, and specificity. Lying and rolling behaviors are present in horses suffering abdominal pain (14), but in donkeys, the frequency of these behaviors is low (1) and they were not noted in this study. In this way, the posture item was excluded after refinement of the scale.

When the miscellaneous behaviors were combined and analyzed together, they demonstrated sensitivity, but no specificity. Although the sum of these behavioral scores decreased after analgesia, some behaviors of this item were never observed, such as rolling, lying down and standing up, digging, and kicking the abdomen. Therefore, they were removed after refinement. Elevation of a pelvic limb presented excellent specificity, and although 23 of the 40 animals demonstrated this behavior at the time of greatest pain, the sensitivity was low. After refinement, this behavior was maintained based on specificity, frequency of occurrence, and responsiveness, and because it has also been described in horses after castration (11) and was the most relevant behavior in the ethogram of donkeys with acute pain in the parallel study performed simultaneously with the present one (2). Exposing the penis and shaking the tail were neither sensitive nor specific. The majority of the animals swung their tail independently of the presence of pain; as this behavior is associated with the attempt to dispatch flies (1, 2), it was excluded from the scale after refinement.

In regard to the frequency and distribution of pain score levels for each item along the time, scores 1 and 2 were more frequent when donkeys were suffering pain, at 3–4 (M1) and 24 h after surgery (M3). The moment of maximum pain (M1) showed a more significant percentage of score 2, compared to the moment of moderate pain (M3), demonstrating the importance of providing two levels of pain score intensities.

Reliability is a prerequisite to validate a scale (16); repeatability was very good and inter-evaluator reliability ranged from moderate to good. Evaluator 3 had the worst reproducibility, probably because he was the least experienced evaluator in managing donkeys. As observed in equines (43) and laboratory animals (44), prior evaluators' training is apparently required, even if the evaluators have experience in equines, to improve inter-evaluator reliability. However, for most evaluators (RE vs. Ev. 1 and 2), inter-evaluator reliability was good in this study even without training. Our results were above that observed in horses after acute abdominal surgery (0.3 ± 0.1) (42), but lower than after orthopedic surgeries (0.8–1) (6).

Although the VAS, NS, and SDS presented responsiveness, their inter-evaluator reliability values were lower than DOPS, as described in horses with abdominal pain (45, 46). These scales depend on the evaluator's experience (14, 47). The use of scales with behavioral indicators described in detail in the form of scores facilitates the evaluation process. It makes it more objective and reliable, providing a more consistent evaluation with less evaluator influence (48). In VAS, NS, and SDS there is also a tendency to avoid assigning scores at the extremities (16).

Criterion validity is the ability of the test to agree well with an assignment it intends to foresee. The classical concept of criterion validity is concurrent validity, based on the correlation between the new instrument against a gold standard one (16) at the same time. In humans, when self-reporting of pain is possible, the VAS may be considered a gold standard method. However, due to the complexity of the phenomenon, even in humans, it is difficult to delineate a gold standard method, with an objective benchmark, to define pain (49). Because self-reporting of pain is impossible in animals, criterion validation requires an alternative methodology. In donkeys, and even in horses, there are no gold standard instruments to compare with a new proposed tool. The previously published studies in donkeys have not performed a complete validation analysis (3); thus, the possible ways to assess criterion validity are comparisons against the instruments available, namely the NR, SDS, and VAS. Although the aforementioned approach may be arguable, it has been used in other species (11, 17, 45, 46, 50), children, and older people in medicine (51, 52). In this context, the proposed scale demonstrated good correlation with the VAS and NS and moderate correlation with the SDS, in the same way as in equines (11, 53).

Other tests were included to compensate for a possible flaw using only the methods mentioned above to assess criterion validity. One of these was to calculate the agreement between the reference and the other evaluators, as reported in other species (8, 11, 17). In this case, agreement ranged from moderate to good. A third approach for testing criterion validity was predictive validation, based on the number of donkeys that would receive rescue analgesia according to the Youden Index in the moment of most significant pain (M1). Because between 80 and 98% of donkeys would receive rescue analgesic in the moment of greatest pain, the instrument was able to predict that donkeys were suffering pain and should hence receive treatment.

The internal consistency was 0.64, which is minimally acceptable. The item values, assessed separately, were analogous and indicated a sufficient relation between the scale items' responses and that the categories of behaviors may similarly measure pain (54). The heteroscedasticity of the behaviors can reduce α, so these values should be interpreted in light of the characteristics of the measure to which they are associated, and of the population where the measurement was performed (55). Otherwise, α values > 0.9 indicate that the instrument is redundant and requires a reduction of items (56). The most widespread scales used for pain evaluation in horses (6, 11, 42, 50, 57) and donkey (3) did not present internal consistency values for comparison purposes.

The item-total correlation, which indicates the importance of each item, and assures scale homogeneity, was within the acceptable limits of 0.3–0.7 (16), except for head position and direction (slightly below 0.3). Values above 0.7 indicate that the scale is too specific, and below 0.3 indicate a lack of homogeneity. Head position and direction were the only items that also showed low load values in PCA. Ear position, reaction to palpation of the wound, and lift pelvic limb are behaviors also presented by horses suffering pain (6, 11, 42, 43, 46). The ears' position was the most relevant behavior for evaluating acute pain in donkeys since it was the only behavior that presented good sensitivity and specificity. This behavior has also been associated with pain in donkeys in previous studies, especially if the ears are facing backward (1, 3, 4, 15). The reaction to palpation of the wound was sensitive to diagnose pain, as observed in the orthopedic pain scale (6) and abdominal pain in horses (42), and is considered one of the most important behaviors in the assessment of visceral pain in equines (50).

The principal component analysis revealed that, except for head direction, which provided little impact for the scale, the items were associated well. Head direction was maintained after refinement as it was specific and responsive.

The high area under the curve observed indicates that the instrument presents excellent discriminatory capacity (39) and high accuracy of the rescue point (41). The recommended score for analgesic intervention directs the provision of analgesic therapy and evaluates intervention's efficacy (58). The cut-off point for rescue analgesic based on the Youden index was ≥ 4, representing 36% of the total value of the scale. Based on the diagnostic uncertainty zone a score <3 indicates “no pain” and > 4 indicates “presence of pain” with a better degree of certainty than the Youden index. However, determination of the intervention analgesic score does not replace the professional's autonomy and clinical experience, so that analgesia for animals with scores <4 should not be neglected if the professional considers it necessary (59). These results are similar to those reported in horses with orthopedic pain (60), with scores of 5–8 for mild pain, 8–10 for moderate pain, and > 10 for severe pain (6), representing 20% (8 of 39) of the total scale. For the Equine Utrecht University Scale for Facial Assessment of Pain (EQUUS-FAP), scores of 3–5 would represent mild pain, 5–8 moderate, and > 8 intense pain (50), representing 28% (5 of 18) of the full scale. For the two recent instruments proposed to assess pain in donkeys (EQUUS-DONKEY-COMPASS and EQUUS-DONKEY-FAP) (3), the mean and maximum 95 percentile scores compared to their total scores in donkeys suffering colic pain were about 17% and below 40% for both instruments, respectively. These previous results in horses and donkeys suggest that some items may be redundant. Therefore, item-by-item analysis could be performed for refinement after a thorough validation of these scales. In our study the maximum values were 10 out of the total score of 11 (91% of the total sum), showing that all behaviors included in the scale are relevant and reflecting the importance of the refinement process.

This study presented some limitations. Although the animals were in good clinical and laboratorial condition, considering they were animals from a rescue society with no definite history, the presence of some chronic clinical conditions, associated mainly with locomotive problems, cannot be excluded. Donkeys in Pakistan presented a high prevalence of lameness and joint disorders (61). This fact may have induced similar points in the scores between M0 and M1 for the categories of locomotion when led and appetite for the food offered, which did not present sensitivity. Concerning locomotion, while horses tend to be restless in stress situations, donkeys usually stand still and are reluctant to move (62). Thus, as the locomotion item does not seem relevant to diagnose pain in donkeys, it was removed from the scale. The same applies to appetite as it was neither specific nor sensitive and did not show responsiveness.

Another limitation was the presence of flies that could influence some behaviors such as shaking the tail and moving the ears (2). Shaking the tail was removed after refinement, and insects apparently did not affect ear position as it showed the best results in all validation criteria. The presence of flies is common in stalls, especially in a tropical weather environment. The effect of the presence of flies on donkeys behavior has been previously investigated in the parallel study (2), which showed that in pain-free donkeys, a dirty stall increased tail, head, and ear movements compared to a clean stall, therefore these behaviors may be confounders when pain is assessed in donkeys in the presence of insects. Still, in the clean stall, there was no apparent difference in tail swishing between pain-free donkeys and in donkeys after surgery, and analgesia did not modify tail swishing either. According to this previous study (2) and to present one tail swishing is not a specific behavior to assess pain in donkeys and maybe a confounder, confirming its exclusion from the final version of the scale.

The in-person evaluator edited the short videos to be assessed by the blind evaluators, as she was familiar with the behaviors. While such short videos do not represent the duration of each behavior observed for 1 h, the editor did her best to assemble and resume the behavior repertoire in the short videos. This approach has been used previously to validate pain scales in dogs (63), cats (17), cattle (8), horses (11), pigs (9), and sheep (10). This method provides data to assess intra- and inter-evaluator reliability and perform calculations to validate the scales. Therefore, DOPS will require in-person validation as reported in cats (7, 17) to guarantee it is a valid instrument for clinical use and to investigate what is the required period for pain assessment in practice.

The fact that the in-person evaluator was the reference evaluator may be considered a limitation of the study. Like in previous studies in animal species (8–10, 17, 60, 64) and children (65), the gold standard evaluators (reference evaluators) were those that performed the in-person pain assessment, and therefore were not blinded. The reference evaluator was chosen according to the following criteria: she was the investigator directly involved in the fieldwork and, therefore, had training and substantial knowledge in assessing pain in donkeys. In addition, she demonstrated the best repeatability among all evaluators and coherence in the results, shown by indication of rescue analgesia in M1 and M0 based on the Youden index. Based on this, the evaluator would demonstrate good ability to differentiate a pain-free state from a painful state. To counterbalance the choice of the reference evaluator, the results from responsiveness showed a great similarity among evaluators.

Hence, the in-person evaluator was not completely blind, however as (i) the order of videos (M0, M1, M2, and M3) was randomized, renamed for each donkey and (ii) rearranged again into a new random sequence at the second evaluation phase, (iii) she assessed a large number of donkeys and videos (160), and (iv) as, unlike horses, donkeys have the same color and are very similar in their appearance, it would be very difficult for the in-person evaluator to identify and remember her in-person assessment against the video analysis.

Although it is generally conceived that donkeys require social contact, they were housed alone during the experimental phase, which might interfere with behavior. This study aimed to mimic a clinical situation when donkeys may be sent to hospitals by themselves, where they are housed in individual stalls. As the donkeys were alone in all moments, including baseline, behavior changes were apparently compared in equal conditions.

A final limitation of the study would be the lack of a negative and positive control group. The potential influence of postoperative sedation on pain behavior could be a concern; however, we do not believe this would be relevant in the current study, as the donkeys were fully recovered at the moment of greatest pain (3–4 h after recovery from anesthesia) since the maximum recovery period was 52 min after the end of anesthesia. A previous study performed in horses included two positive control groups (anesthesia and anesthesia plus analgesia) using a similar pain scale (11). In that study, neither anesthesia nor anesthesia plus analgesia without surgery had any effect on pain scores. Lastly, baseline measurements would partially compensate for the limitation regarding the lack of a negative control group.

## Conclusions

Acute pain can be diagnosed and quantified in donkeys submitted to castration using the DOPS, since the instrument presented very good repeatability, good reproducibility, responsiveness for all individual items (construct validity), content and criterion validity, internal consistency, adequate item-total correlation, association among most items, and excellent accuracy for rescue analgesic point.

## Data Availability Statement

The original contributions presented in the study are included in the article/Supplementary Material, further inquiries can be directed to the corresponding author/s.

## Ethics Statement

The animal study was reviewed and approved by Ethics Committee on Animal Use of UFERSA (Universidade Federal Rural do Semi-árido). Written informed consent was obtained from the owners for the participation of their animals in this study.

## Author Contributions

MO data acquisition, interpretation, and drafting and writing the manuscript. IL, AM, LM, and TN data acquisition and interpretation. PT statistical analysis, data interpretation, and presentation. SL and VP conception and design, data interpretation, and drafting and final writing of the manuscript. All authors contributed to the article and approved the submitted version.

## Conflict of Interest

The authors declare that the research was conducted in the absence of any commercial or financial relationships that could be construed as a potential conflict of interest.
